# Transmembrane Interactions of Full-length Mammalian Bitopic Cytochrome-P450-Cytochrome-b_5_ Complex in Lipid Bilayers Revealed by Sensitivity-Enhanced Dynamic Nuclear Polarization Solid-state NMR Spectroscopy

**DOI:** 10.1038/s41598-017-04219-1

**Published:** 2017-06-23

**Authors:** Kazutoshi Yamamoto, Marc A. Caporini, Sang-Choul Im, Lucy Waskell, Ayyalusamy Ramamoorthy

**Affiliations:** 1 Biophysics and Department of Chemistry, University of Michigan, Ann Arbor, MI 48109-1055 USA; 2 Bruker Biospin Corporation, 15 Fortune Drive, Billerica, MA 01821 USA; 3 Department of Anesthesiology, VA Medical Center, University of Michigan, Ann Arbor, MI 48105 USA

## Abstract

The dynamic protein-protein and protein-ligand interactions of integral bitopic membrane proteins with a single membrane-spanning helix play a plethora of vital roles in the cellular processes associated with human health and diseases, including signaling and enzymatic catalysis. While an increasing number of high-resolution structural studies of membrane proteins have successfully manifested an in-depth understanding of their biological functions, intact membrane-bound bitopic protein-protein complexes pose tremendous challenges for structural studies by crystallography or solution NMR spectroscopy. Therefore, there is a growing interest in developing approaches to investigate the functional interactions of bitopic membrane proteins embedded in lipid bilayers at atomic-level. Here we demonstrate the feasibility of dynamic nuclear polarization (DNP) magic-angle-spinning NMR techniques, along with a judiciously designed stable isotope labeling scheme, to measure atomistic-resolution transmembrane-transmembrane interactions of full-length mammalian ~72-kDa cytochrome P450-cytochrome b_5_ complex in lipid bilayers. Additionally, the DNP sensitivity-enhanced two-dimensional ^13^C/^13^C chemical shift correlations via proton driven spin diffusion provided distance constraints to characterize protein-lipid interactions and revealed the transmembrane topology of cytochrome b_5_. The results reported in this study would pave ways for high-resolution structural and topological investigations of membrane-bound full-length bitopic protein complexes under physiological conditions.

## Introduction

Bitopic membrane proteins with a single membrane-spanning α-helix represent more than half of all membrane proteins, which consist of approximately one third of all open reading frames (ORFs), in human genomes^1–3^. Their dynamic protein-protein and protein-ligand interactions in the membrane environment play a plethora of vital roles in the cellular processes associated with human health and diseases, including cell signaling, cell morphology regulation, and enzymatic catalysis^3^. Hence, bitopic proteins are considered to be one of the most promising pharmaceutical targets^3^. In particular, numerous signal transductions of bitopic receptors can be activated predominantly through molecular interactions between transmembrane domains, based on their conformational changes, homo-/heteromeric-associations, and their formations of signaling platforms^3^. The high-resolution structures and dynamics of these complexes in lipid bilayers are crucial to fully elucidate their biological functions^1–3^. Notwithstanding the recent advances in biophysical techniques^4–10^, these bitopic proteins and their complexes still pose tremendous challenges for atomic-level structural determinations by X-ray crystallography^11–14^ and conventional solution nuclear magnetic resonance (NMR) spectroscopy^10, 15–19^. These challenges arise from (i) difficulties to express, solubilize, and purify the hydrophobic domains of proteins, (ii) obstacles with obtaining suitable and stable environments for crystallization or traditional solution NMR spectroscopy, (iii) disordered features of lipid bilayers to form ordered crystallizations, and (iv) the colossal molecular sizes of proteins with a membrane environment and their slow overall tumbling motions for the standard solution NMR methodologies. These are particularly notable drawbacks for bitopic proteins that contain bulky soluble domains, such as the membrane-anchored catalytic enzymes like mammalian cytochrome P450, cytochrome b_5_, and cytochrome P450 reductases^15, 20–24^. Due to the aforementioned challenges, only structures of soluble domain fragments of these single-pass membrane proteins are predominately available in the Protein Data Bank^25–28^. For a complete understanding of their active interactions, it is crucial to obtain the structural information of the biologically active *full-length* forms with both transmembrane and extramembranous domains, especially in the case of membrane-bound cytochrome P450 complexes^15, 20–22, 24^. However, only a few biophysical techniques are capable of probing lateral interactions and assemblies of bitopic proteins in the lipid bilayers. Experimentally, such transmembrane interactions between these single membrane-spanning proteins can be investigated using traditional biophysical techniques, including SDS-PAGE, sedimentation equilibrium analytical ultracentrifugation, fluorescence resonance energy transfer (FRET), crosslinking, and cellular membrane reporter assays. SDS-PAGE^29, 30^ and sedimentation equilibrium analytical ultracentrifugation^31, 32^ can be applied to membrane protein complexes in micelles as a membrane mimetic medium. Although these two methods do not require significant modifications nor high concentrations of proteins, yet they can give rise to an array of potential difficulties, including experimental artifacts due to the properties of strong destabilizing detergents, weak site-specific transmembrane interactions, altered structures in high-curvature micelles, and/or the large stoichiometry of transmembrane proteins with micellar aggregates;^29, 30, 33^ both methods are additionally unsuitable for cellular settings^33^. On the other hand, FRET^30, 34, 35^, crosslinking^36^, and reporter assays in biological membranes^37–39^ can be performed in *in-cell* conditions, albeit they require significant modifications in targeted proteins, which can also cause potential errors in the measurements arising from the alternations of protein properties, and/or the complexity of biomolecular interactions in *in-vivo* conditions. These facts collectively suggest that multiple independent biophysical techniques are necessary to confirm the site-specific interactions of membrane proteins^30, 33, 40^. Furthermore, none of these methods can reveal detailed high-resolution structural information of the interactions between bitopic proteins in their full-length form. It is therefore essential to develop biophysical approaches and methodologies that enable us to investigate the molecular interactions of functional full-length membrane protein structures embedded in lipid bilayers at atomic level resolution. In this context, solid-state NMR (ssNMR) spectroscopy is particularly a powerful method for providing a detailed atomic-resolution structural information of the dynamic protein-protein and protein-ligand interactions in a lipid bilayer environment^41–45^. In this study, we demonstrate the feasibility of ssNMR spectroscopy^46–49^ for probing transmembrane interactions of membrane-bound bitopic proteins, namely the intact mammalian cytochrome P450-cytochrome b_5_ complex, in lipid bilayers.

Cytochromes P450 are a ubiquitous superfamily of mixed-function monooxygenases found in all kingdoms of life with highly conserved functions in eukaryotes^50, 51^. Membrane-bound microsomal cytochromes P450 play a central role in the metabolic clearance of various exogenous and endogenous compounds in the liver to reduce toxic exposure in human bodies, which include approximately 75% of the drugs in current clinical use^50, 51^. As a key step in their catalytic cycles, their oxidation of substrates requires two electrons to be transferred to the heme-containing reaction center of cytochromes P450 from their redox partners, such as cytochromes b_5_, and cytochrome P450 reductases^23, 50, 51^. Each of the three microsomal cytochromes possesses a single transmembrane α-helix, which is vital for their enzymatic activities^20, 23, 50, 51^. This crucial transmembrane-transmembrane interaction of the full-length liver microsomal cytochrome P450 2B4 with its full-length redox partner, cytochrome b_5_, is investigated in this study.

Rabbit cytochrome b_5_, a 16.7 kDa protein, is composed of three structurally distinct domains: a 25-amino acid transmembrane α-helix domain at the carboxyl-terminus and a 95-residue heme-containing electron-carrier soluble domain at the amino-terminus; the two domains are connected via a 14-amino acid flexible linker domain^22, 23, 51^. In addition to the aforementioned drug metabolisms through the complex formations, cytochrome b_5_ also catalyzes a wide variety of biosynthesis including testosterones, cholesterols, and unsaturated lipids^51^. Depending on the substrates, the cytochrome P450 isozymes and/or the experimental conditions, cytochrome b_5_ can significantly enhance the enzymatic turnover of cytochrome P450 by up to 100-fold, in some cases, however, cytochrome b_5_ does not affect or even inhibits the catalytic activities of cytochromes P450. In the case of rabbit cytochrome P450 2B4, a 55.7 kDa protein, when metabolizing benzphetamine and methoxyflurane stoichiometrically, cytochrome b_5_ stimulates the enzymatic activities of cytochrome P450 2B4 predominantly by reducing the amount of the side-product superoxide^51, 52^. In order to fully understand the molecular mechanism of these key roles of cytochrome b_5_, elucidating the atomic-resolution structure of the full-length form of cytochrome P450-cytochrome b_5_ complex is quintessential. Despite the physiological importance of their full-length forms for their complete catalytic functions,the X-ray and solution NMR structures of both membrane-bound eukaryotic cytochrome P450 and cytochrome b_5_ have been determined only for the truncated forms of hydrophilic heme-containing cytosolic catalytic domains, in which the hydrophobic transmembrane domains were removed to overcome the difficulties in overexpression, solubilization, purification, and crystallization^25, 53^, with the exception of recent solution- and solid-state NMR studies from our group on full-length rabbit cytochrome b_5_ and cytochrome P450 2B4^15, 20–22^, as well as an X-ray crystallography study on cytochrome P450 51A1^11^. These high-resolution full-length structures are significant breakthroughs towards the complete understanding of the functional aspects of membrane-bound mammalian cytochromes, as it is known that the lack of transmembrane anchors reduces to only 40% of all enzymatic activities in the case of cytochrome P450 2B4, and results in a total loss of electron transfer capability to cytochromes P450 in the case of cytochrome b_5_^15, 20–22^. A recent solution NMR study from our group revealed the first full-length dynamic interactions of the membrane-bound complex between rabbit cytochrome P450 2B4 and cytochrome b_5_ reconstituted in isotropic bicelles and the electron transfer pathway between catalytic domains of the complex. However, these traditional solution NMR techniques could not have explained the importance of the transmembrane domains for the catalytic activities of the cytochrome P450 complex due to the experimental limitations of solution NMR spectroscopy, which are attributed to the intermediate time scale of dynamics of transmembrane helices^15^. Here, we demonstrate the use of state-of-the-art sensitivity enhancement by dynamic nuclear polarization (DNP) NMR spectroscopy under magic angle spinning (MAS)^54–62^ and a judiciously designed isotope labeling scheme to overcome the difficulties associated with the structural studies of full-length bitopic proteins. The combination of these developments have enabled us to report the DNP solid-state NMR spectroscopy probing transmembrane-transmembrane interactions of the cytochrome P450-cytochrome b_5_ complex embedded in lipid bilayers at an atomic-level for the first time.

## Materials and Methods

### Materials

Uniformly-deuterated [D_8_] glycerol, deuterium oxide, [1-^13^C] valine, [2-^13^C] leucine, [3-^13^C] alanine, and tryptophan (indole ring-2-^13^C) were purchased from Cambridge Isotope Laboratories, Inc. (Andover, MA). DNP polarizing agents, AMUPol^63^ and TOTAPOL (1-(TEMPO-4-oxy)-3-(TEMPO-4-amino)propan-2-ol)^64^, were kindly provided by Bruker Biospin. DMPC (1,2-dimyristoyl-*sn*-glycero-3-phosphocholine) was purchased from Avanti Polar Lipids, Inc. (Alabaster, AL). All other chemicals were purchased from Sigma-Aldrich (St. Louis, MO).

### Sample preparation of selectively ^13^C-labeled cytochrome b_5_ and ^13^C-labeled cytochrome b_5_ –uniformly ^15^N-labeled cytochrome P450 complex incorporated into multilamellar lipid vesicles with DNP polarizing agents

Stock biradical solutions of [D_8_]glycerol/D_2_O/H_2_O (60:30:10 volume ratio) containing 40 mM DNP polarizing agents (AMUPol, or TOTAPOL) and a DNP solution of [D_8_]glycerol/D_2_O/H_2_O (60:30:10 volume ratio) were prepared and kept in a -80 °C deep freezer. Brief sonication was applied to dissolve TOTAPOL powder in the stock solution. However, sonication was not necessary to dissolve AMUPol powder due to its high aqueous solubility. The overexpression and purification of selectively ^13^C-labeled cytochrome b_5_ and uniformly ^15^N-labeled cytochrome P450 were reported previously^15, 22, 52^. Solution NMR measurements on ^13^C-labeled cytochrome b_5_ embedded in *q* = 0.25 DLPC/DHPC isotropic bicelles confirmed that the scrambling of isotope ^13^C-labels did not occur. Five milligrams of DMPC powder was hydrated using 10 μl of 2.98 mM selectively ^13^C-labeled cytochrome b_5_ in a DNP solution (or 10 μl of 1.2 mM selectively ^13^C-labeled cytochrome b_5_-uniformly ^15^N-labeled cytochrome P450 complex in a DNP solution), [D_8_]glycerol, and stock biradical solutions. The resulting samples were homogeneously mixed by vortexing, followed by the application of freeze-and-thaw cycles for five times. The selectively ^13^C-labeled cytochrome b_5_ (or selectively ^13^C-labeled cytochrome b_5_-uniformly ^15^N-labeled cytochrome P450 complex) reconstituted in DMPC multilamellar vesicles with a 10 mM DNP polarizing agent and were packed into 3.2 mm sapphire MAS rotors. It is known that approximately 10 mM of biradicals, including AMUPol and TOTAPOL, is sufficient to achieve the optimum DNP enhancements. The NMR probe was pre-cooled to 99.5 K prior to inserting the samples into the probe.

### NMR measurements

DNP-enhanced ssNMR experiments^65–67^ were performed on an Avance III 600-MHz Bruker NMR spectrometer equipped with a 395.18 GHz second-harmonic gyrotron with a 3.2 mm ^1^H, ^13^C, ^15^N triple-resonance low-temperature MAS probe at 99.5 K. The MAS rates were set to 8.5 and 12.5 ± 0.003 kHz for one- and two-dimensional experiments, respectively. The samples were irradiated with 9 W of CW microwave power for DNP experiments. The sample temperature was calibrated by *T*_*1*_ spin-lattice relaxation measurements of KBr under microwave irradiation and MAS. The DNP signal enhancement factors (*ε*) were obtained by comparing the peak intensities from spectra acquired with and without microwave irradiations, by keeping all other experimental conditions identical. The recycle delays were set to 1.3 × *T*_*1*_ (spin-lattice) relaxation time for each sample, which provided the optimum sensitivity. The ^13^C NMR spectra in Supplementary Figure S1(B and C) were collected at 273 K on a 600-MHz Varian/Agilent solid-state NMR spectrometer using a 3.2 mm triple-resonance electric-field-free BioMAS probe. The ^13^C NMR chemical shifts were referenced with respect to 4,4-dimethyl-4-silapentane-1-sulfonic acid (DSS) using adamantane as an external reference.

## Results and Discussions

### Optimization of polarizing agents for the sensitivity enhancement by DNP-NMR on full-length membrane-bound cytochromes

Rabbit cytochrome b_5_ and cytochrome P450 were overexpressed and purified from *E. Coli* ^68^, and then reconstituted^20, 22, 69^ into 1,2-dimyristoyl-*sn*-glycero-3-phosphocholine (DMPC) lipid bilayers hydrated with biradical solutions of [D_8_]glycerol/D_2_O/H_2_O (60:30:10 volume ratio) containing DNP polarizing agents, AMUPol^63^ or TOTAPOL (1-(TEMPO-4-oxy)-3-(TEMPO-4-amino)propan-2-ol)^64^, as described in the previous section. The production of membrane-bound mammalian cytochromes P450 is quite challenging because of their instability and extensive hydrophobicity. In fact, the yield of isotopically labeled full-length cytochrome P450 is extremely low, which is unfavorable for traditional ssNMR-based structural studies which generally require large sample quantities.

**Figure 1:**
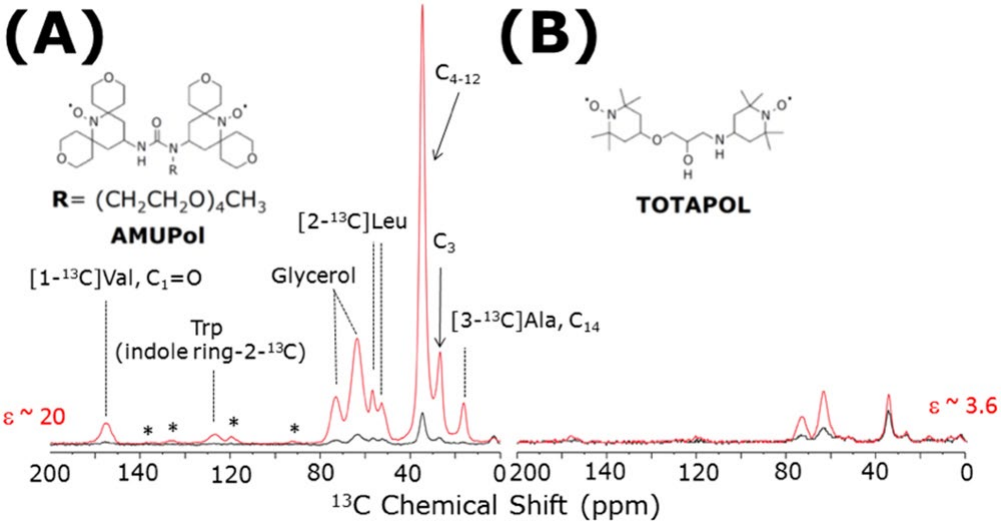
Higher Dynamic Nuclear Polarization (DNP) efficiency of the AMUPol polarizing agent. One-dimensional ^13^C CPMAS NMR spectra of selectively ^13^C-labeled cytochrome b_5_ incorporated in DMPC MLVs with microwave irradiation on (red), and off (black). Cytochrome b_5_ was selectively ^13^C-labeled with [1-^13^C] valine, [2-^13^C] leucine, [3-^13^C] alanine, and tryptophan (indole ring-2-^13^C), as discussed in the main text. Chemical structures of biradical polarizing agents are shown in (**A**) AMUPol, and (**B**) a conventional polarizing agent, 1-(TEMPO-4-oxy)-3-(TEMPO-4-amino)propan-2-ol (TOTAPOL) as insets. **(A)** 10 mM AMUPol, or **(B)** 10 mM TOTAPOL in [D_8_]glycerol/D_2_O/H_2_O (60/30/10 volume ratio) were used as DNP polarization agents. The signal enhancements (*ε*) of ~20 with 10 mM AMUPol, and ~3.6 with 10 mM TOTAPOL, as described in Table 1. The samples were spun at 8.5 kHz MAS, and sample temperatures were kept at 99.5 K. A CP contact time of 0.8 ms, a 100 kHz SPINAL64 pulse sequence^101^ to decouple protons during the signal acquisition time of 25.9 ms, and a recycle delays of 3.9 s (for 10 mM AMUPol, A), and 3.3 s (for 10 mM TOTAPOL, B) were used. Total experimental time was 3 min. Spinning side bands are indicated by asterisks in the spectra. A background signal arising around 0 ppm is from the silicon rubber seal used in MAS rotors.

Furthermore, our previous studies revealed that multidimensional MAS ssNMR experiments performed on uniformly ^13^C-labeled cytochrome b_5_ reconstituted in DMPC mutilamellar vesicles (MLVs) gave rise to spectra with low signal-to-noise ratio at 310 K^69^. This surprisingly poor performance of dipolar-based ssNMR is attributed to the highly dynamic features of cytochrome b_5_^69, 70^. In this study, we demonstrate that the sensitivity enhancement afforded by DNP spectroscopy at cryogenic temperatures can overcome both the interference with molecular motions and the low sensitivity. In the DNP experiments presented here, samples were prepared with two different DNP polarizing agents, AMUPol^63^ and TOTAPOL^64^, as described in the Materials and Methods section. In order to evaluate the polarizing efficiency of these two different biradicals, one-dimensional ^13^C CPMAS NMR spectra^71^ of selectively ^13^C-labeled cytochrome b_5_ embedded in DMPC MLVs were recorded with and without microwave irradiation, as shown in Fig. 1(A and B). These spectra indicate that the high efficiency of the DNP effect and the suppressed molecular motions at frozen conditions both contribute to excellent sensitivity enhancements of these CPMAS spectra. On the other hand, the undesired line-broadenings in the spectra were also observed because of restricted molecular motions and/or the conformational heterogeneity at cryogenic temperatures, as discussed in previous studies^70, 72^. However, the ^13^C chemical shift resonances from the selectively labeled sites of proteins do not overlap significantly with the DMPC lipid bilayers or glycerol ^13^C signals due to the careful choice of the labeling sites, which results in highly resolved and well dispersed ^13^C resonances. Particularly, Trp (indole ring-2-^13^C) resonances around 125 ppm are completely isolated from other signals from protein, glycerol and lipid (Fig. 1A).

As shown in Fig. 1 and Table 1, our experiments yielded DNP enhancement factors (*ε*) of ~20 and ~3.6 with 10 mM AMUPol and 10 mM TOTAPOL, respectively. These DNP enhancements of AMUPol and TOTAPOL are comparable with previously reported values on non-crystalized membrane proteins in lipid bilayers^56, 58, 60, 73^. Remarkably, AMUPol produced higher DNP enhancement for all of the ^13^C resonances, with an average of about 7.3 times higher signal enhancements, as reported in Table 1. This observation agrees with previous studies on the comparison of AMUPol and TOTAPOL using model compounds and membrane proteins in cellular settings^63, 73^. The higher DNP efficiency of AMUPol is ascribed to its higher solubility, optimum electron relaxation time, large electron-electron dipolar coupling, and rigid form of the chemical structure^63^. Practically, AMUPol maintains its superb performance at high magnetic fields, as well as under fast MAS speeds up to 14 kHz^63^. Among various cross-effect DNP polarizing agents^74–78^, AMUPol would thus be one of the most suitable biradicals for biological applications up-to-date. On the other hand, the most commonly used DNP biradical, TOTAPOL, has some disadvantages on its poor performance at higher magnetic fields, and faster MAS speed (>3 kHz), which can still be useful for studies on materials^64^. In light of these results, AMUPol was our obvious choice as a polarizing agent in our further DNP-based experiments on the protein systems used in this study. Although DNP spectroscopy can drastically increase the signal-to-noise ratio of NMR spectra, significant line-broadening at low temperatures can be a fatal problem in these experiments^70, 72^. In order to fully exploit the promising advantage of sensitivity-enhanced DNP-ssNMR spectroscopy, the strategic isotope labeling of proteins is therefore necessary to improve the spectral resolution at frozen conditions.

**Table 1:** Proton spin-lattice relaxation (*T*_*1*_) times and DNP enhancements obtained from selectively ^13^C-labeled cytochrome b_5_.

^13^C chemical shift (ppm)	176	120	64	57	35	27	17
	(C=O)	(C^δ1^-Trp)	(glycerol)	(C_α_-Leu)	(C_4-12_)	(C_3_)	(C_14_)
^1^H T_1_ with 10 mM AMUPoL (s)	3	1.4	2.5	1.5	3	2.8	2.5
ε with 10 mM AMUPoL	10	8	12	10	15	15	20
^1^H T_1_ with 10 mM TOTAPOL (s)	1.4	N/A	2.4	1.8	1.3	1.1	2.5
ε with 10 mM TOTAPOL	2.3	1.8	4	1	1.5	1.4	2

Proton *T*_*1*_ values obtained from selectively ^13^C-labeled cytochrome b_5_ reconstituted into DMPC MLVs with polarizing agents (10 mM AMUPol, or 10 mM TOTAPOL) in [D_8_]glycerol/D_2_O/H_2_O (60/30/10 volume ratio) using proton saturation recovery with ^13^C CP detection, and the signal enhancement (*ε*) of ^13^C CPMAS spectra obtained from Fig. 1. The ^1^H *T*_*1*_ values were measured for selected ^13^C resonances at 8.5 kHz MAS, 99.5 K sample temperature, and using 8 scans. Errors estimated for the reported *T*_*1*_ values range from 0.001 to 0.03 s. Relaxation times measured without a DNP label are given in Supplementary Table S1.

### Judicious isotope labeling provides well-resolved cross peaks in 2D ^13^C-^13^C chemical shift correlation of membrane-bound cytochrome b_5_

**Figure 2:**
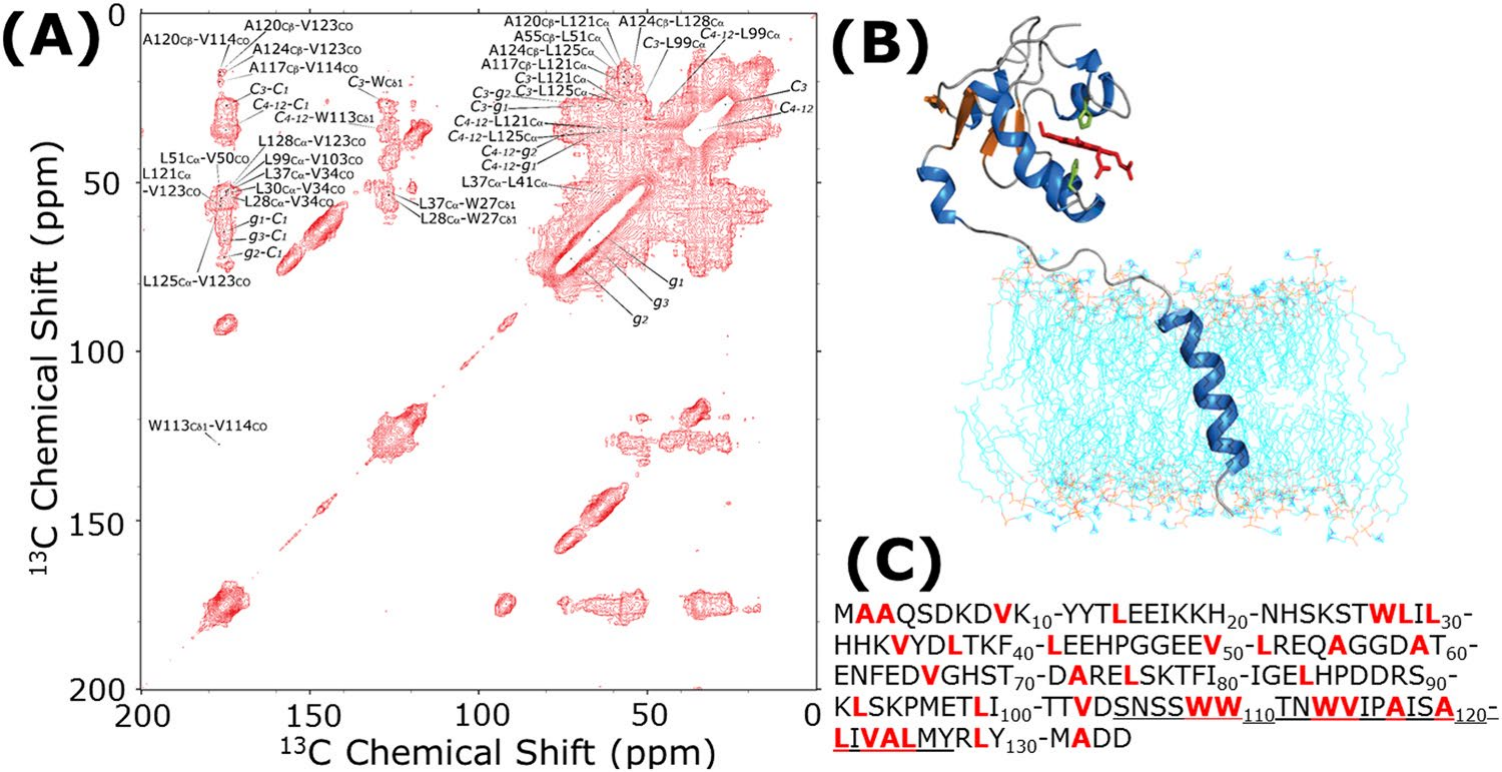
DNP sensitivity-enhanced two-dimensional ^13^C-^13^C chemical shift correlation spectrum of selectively ^13^C-labeled cytochrome b_5_ incorporated into DMPC bilayers with a high efficient polarizing agent, AMUPol. **(A)** Two dimensional ^13^C-^13^C proton driven spin diffusion (PDSD) chemical shift correlation spectrum of selectively ^13^C-labeled cytochrome b_5_ embedded in DMPC mutilamellar vesicles with 10 mM AMUPol in [D_8_]glycerol/D_2_O/H_2_O (60/30/10 volume ratio) with microwave irradiation at 12.5 kHz MAS, 99.5 K sample temperature. Cytochrome b_5_ was selectively ^13^C-labeled with [1-^13^C] valine, [2-^13^C] leucine, [3-^13^C] alanine, and tryptophan (indole ring-2-^13^C). A 3 s PDSD mixing time, 128 *t*_*1*_ increments, 32 scans, 4 dummy scans, and 3.9 s recycle delay were used. Total experimental time was 7.9 hours. The CP contact time was 1.5 ms and 100 kHz SPINAL64 pulse sequence was used to decouple protons during the signal acquisition time of 13 ms. Covariance NMR was used for the two-dimensional spectrum processing^102^. (**B**) High-resolution NMR structure of full-length rabbit cytochrome b_5_ in lipid bilayers. As mentioned in the main text, cytochrome b_5_ is composed of three distinct domains, including a heme-containing catalytic soluble domain, a flexible linker domain, and a hydrophobic single-pass transmembrane α-helix. **(C)** The full-length amino acid sequence of rabbit cytochrome b_5_. The underlined area is the hydrophobic α-helix transmembrane domain of the bitopic membrane protein. Positions of selectively ^13^C-labeled amino acids are highlighted in red in the sequence.

Sensitivity-enhanced DNP two-dimensional (2D) ^13^C-^13^C chemical shift correlation experiments under MAS conditions were performed on 10 mM AMUPol-containing membrane-bound selectively ^13^C-labeled cytochrome b_5_ using the proton driven spin diffusion (PDSD) pulse sequence^79, 80^. As reported in our previous studies, 2D ^13^C/^13^C PDSD chemical shift correlation experiments on uniformly ^13^C^15^,N-labeled cytochrome b_5_ at cryogenic temperatures exhibited inhomogeneous broadening of resonances mainly due to the presence of multiple conformations, which largely gave rise to unresolved cross peaks in the all ^13^C/^13^C PDSD spectra at 100 K^70, 73^. This common problematic disadvantage is intrinsically present in various biological samples, even in well-ordered crystalline proteins^72^. In order to achieve better spectral resolution, 2D DNP NMR was performed on cytochrome b_5_ with site-specific ^13^C-labeled chemical groups in selected amino acid residues, as shown in Fig. 2(A). For this experiment, a long mixing time (~3 s) of the PDSD pulse sequence was required to detect long-range distances between the ^13^C spins in this selectively labeled protein. In this spectrum, the observed ^13^C spin pairs in PDSD correlations for the transmembrane α-helix domain were, on average, ~ 5.5 Å apart. This dilution of observed correlations in 2D PDSD experiments allowed us to acquire well-resolved cross peaks of cytochrome b_5_ even at 99.5 K. Furthermore, based on these well-resolved 2D ^13^C/^13^C chemical shift correlations, 80% of ^13^C chemical shift resonances of ^13^C-labeled sites in transmembrane domain can be assigned, as shown in Fig. 2(A) and summarized in Table 2. The chemical shifts of these assigned resonances indicate that the secondary structure of this transmembrane α-helix region is consistent with our previous reports^15, 22^, even at low temperatures, as reported in Table 2. In the previous studies, high-resolution structural interactions between the soluble domains of the cytochrome P450-cytochrome b_5_ complex have been investigated, and structure of the complex was reported^15, 20^. Whereas the atomic-level elucidation of essential physiological interactions between transmembrane domains of this complex is the main focus of this study.

**Table 2:** The secondary structure of the transmembrane region of cytochrome b_5_ determined by DNP sensitivity-enhanced ssNMR spectroscopy.

Amino Acid	Experimental Chemical Shift (*δ*exp, ppm)	Chemical Shift Difference (Δppm = *δ*coil − *δ*exp, ppm)	Secondary Structure
V114 CO	177	−1	α-helix
A117 Cβ	20	−2	α-helix
A120 Cβ	17	2	α-helix
L121 Cβ	57	−2	α-helix
L123 CO	177	−1	α-helix
A124 Cβ	19	1	α-helix
L125 Cβ	57	−2	α-helix
L128 Cβ	55	0	random coil

TT**V**DS-NSS**WW**_110_-TN**WV**I-P**A**IS**A**_120_-**L**I**VAL**-MYR**L**Y_130_-M**A**DD.

The secondary structure of transmembrane regions of cytochrome b_5_ determined by DNP sensitivity-enhanced ssNMR spectroscopy using selectively ^13^C-labeled cytochrome b_5_ incorporated into DMPC bilayers with AMUPol at 99.5 K sample temperature; assigned chemical shifts of selectively ^13^C-labeled cytochrome b_5_ incorporated into DMPC bilayers are given in Supplementary Table S2 and that of DMPC are given in Supplementary Table S3. The C-terminal α-helix transmembrane domain of amino acid sequence of rabbit cytochrome b_5_ is shown at the bottom of the Table. The region 105–127 is the hydrophobic α-helical transmembrane region.Selectively ^13^C-labeled amino acids are highlighted in bold in the amino acid sequence, and assigned ^13^C resonances in these labeled amino acids are underlined. The ^13^C resonances were assigned utilizing the two dimensional ^13^C-^13^C PDSD chemical shift correlation spectrum shown in Fig. 2A; the reported chemical shift values are within an error of ~0.5 ppm as the use of selective labeling enabled a better measurement of the peak position in the spectra, while the use of single amino acid labeled protein in DNP experiments could further assist in the resonance assignment by overcoming the overlapping resonances; but, this would be very expensive, particularly for membrane-associated proteins for which the yield is typically low and the production cost is very high. The secondary structure-dependence of empirical ^13^C chemical shifts were obtained from a previous report^103^.

For the association of transmembrane helices in membrane-associated proteins and their complexes, it has been known that a specific glycine-containing sequence (GxxxG) can facilitate transmembrane α-helix dimerization in a “ridges-into-grooves” manner^33, 40, 81^. Similarly, it has been suggested that the replacement of both glycines in the GxxxG motif with Leu (LxxxL), or Ala (AxxxA) can also promote transmembrane dimerizations in membrane environments^33, 40, 82–84^. Particularly, leucines, which are separated by an i, i + 3 or i, i + 4 pattern, are frequently abundant in membrane proteins as leucine zipper-like motifs^33^. In addition to monitoring these important interaction motifs in the transmembrane α-helix, a site-specific ^13^C isotope labeling scheme was carefully designed so that the ^13^C chemical shifts can be sufficiently dispersed. The five main goals were targeted by adopting this isotope labeling scheme: (1) to obtain higher-resolution spectra at cryogenic temperatures; (2) to assign these ^13^C resonances of proteins efficiently, employing the unique sequence patterns in the amino acid sequence, solely based on the ^13^C/^13^C chemical shift correlation; (3) to utilize assigned ^13^C resonances to estimate the secondary structural information of the membrane proteins through ^13^C chemical shifts of Cα, C_β_, and carbonyl sites; (4) to monitor the aforementioned transmembrane dimerization motifs by labeling important residues, including Leu and Ala; and (5) to probe the structural information on the important anchoring amino acid residues, such as tryptophan, located near the interfacial region of lipid bilayers. To address these goals, we strategically chose specific site labeling of selective amino acids, including [1-^13^C] valine, [2-^13^C] leucine, [3-^13^C] alanine, and tryptophan (indole ring-2-^13^C), to make use of the unique patterns in the amino acid sequence of cytochrome b_5_. In cytochrome b_5_, the unique sets of amino acids, Trp_113_Val_114_, Ala_120_Leu_121_, and Val_123_Ala_124_Leu_125_, are only found in the transmembrane region. These unique geometries of ^13^C labeled positions could give distinctive sets of cross peaks in ^13^C/^13^C chemical shift correlation spectrum, which can readily enable the assignment of the spatial connectivity of ^13^C spins. Therefore, these unique sets of cross peaks can be used as the starting points for signal assignments, and can be followed by a sequential assignment of ^13^C resonances in neighboring amino acids, unless the latter are located in the crowded regions of the lipid bilayers, and/or aliphatic resonances. As previously mentioned, Trp (indole ring-2-^13^C) resonances around 125 ppm are completely isolated from other signals in the 1D NMR spectra. In fact, these Trp residues in transmembrane α-helices are found in the interfacial region of lipid bilayers, and have been considered as anchors stabilizing the helices into the membrane^85–88^. Therefore, Trp residues can be excellent probes to detect lipid-protein interfaces, in order to obtain topological information of the transmembrane domain, despite that broadened cross peaks in the Trp (indole ring-2-^13^C) region around 125 ppm are observed. This signal broadening is primarily due to the multiple conformations of Trp side chains in the lipid bilayers^85, 86, 89^. Nevertheless, excellent sensitivity enhancements rendered by DNP and low temperature, combined with our strategic selectively-labeled proteins, can be successfully used to determine structures and/or to measure interatomic interactions of biomolecules.

### Probing protein-lipid interactions from 2D ^13^C/^13^C chemical shift correlation

In addition to protein-protein interactions in a membrane environment, interatomic interactions between a membrane protein and lipids also play important roles in a variety of cellular activities, including signal transductions, transport, and biogenesis^90^. However, the atomic-level biophysical characterization of protein-lipid interactions presents enormous challenges owing to their inherent complexities, by which both lipids and proteins can mutually affect each other in various ways, such as changes in the backbone conformation, topology, and side-chain orientations^90^. In this study, the high sensitivity of DNP spectroscopy allows us to observe protein-lipid intramolecular interactions between cytochrome b_5_ and lipids.

**Figure 3:**
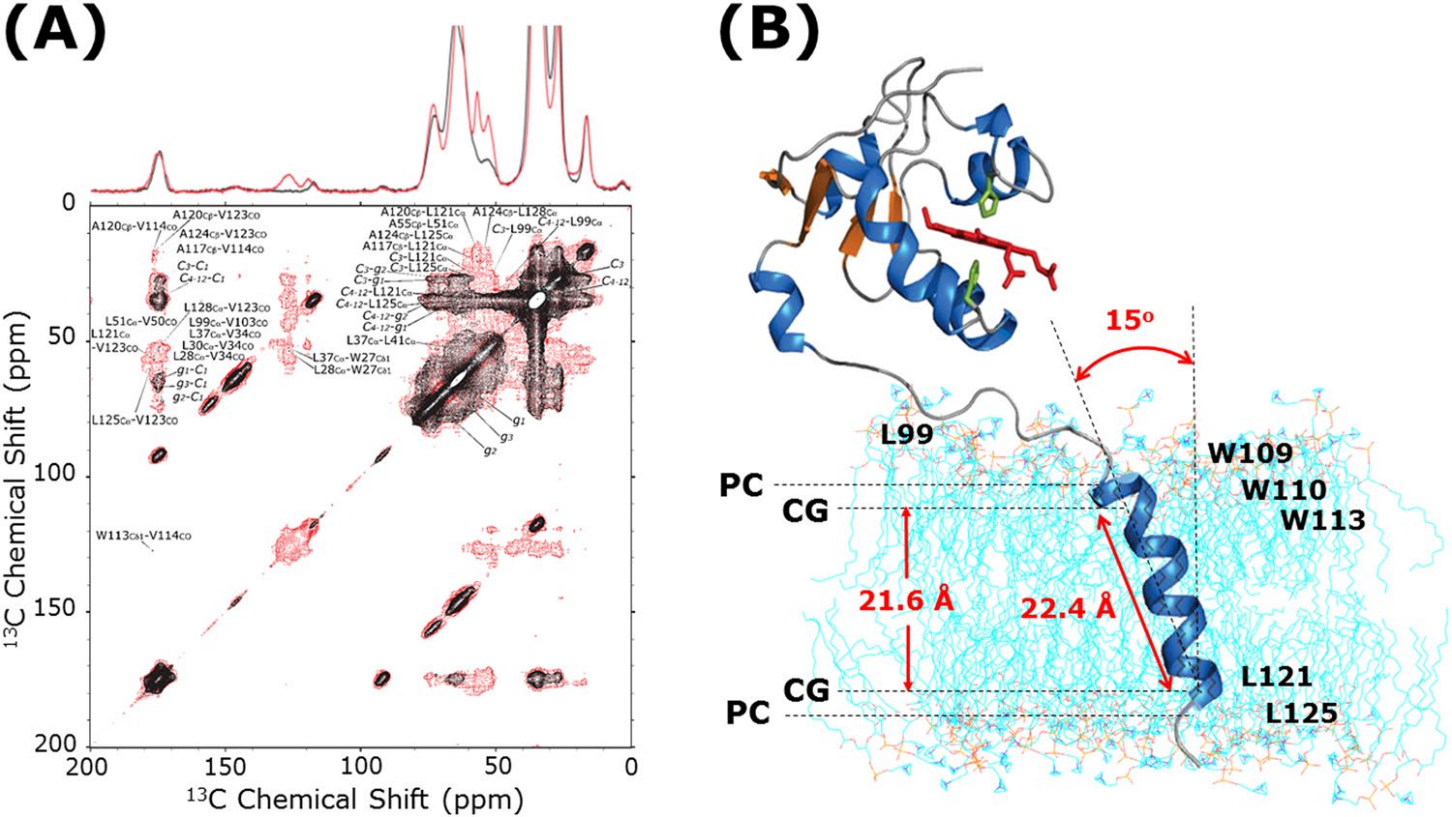
DNP sensitivity-enhanced two-dimensional ^13^C-^13^C chemical shift correlation spectra reveal the atomic-level protein-lipid interactions and topology of transmembrane α-helix of cytochrome b_5_. **(A)** DNP sensitivity-enhanced two dimensional ^13^C-^13^C PDSD chemical shift correlation spectrum of selectively ^13^C-labeled cytochrome b_5_ embedded in DMPC MLVs from Fig. 2(A) (in red) is overlaid on the ^13^C-^13^C PDSD chemical shift correlation spectrum of 50%(w/v) DMPC bilayers from Supplementary Figure S2(A) (in black). A 3 s PDSD mixing time, MAS spinning speed of 12.5 kHz, and 99.5 K sample temperature were used. Detailed experimental conditions are described in the captions of Fig. 2(A) and Supplementary Figure S2(A). (**B**) Protein-lipid interaction sites of the full-length rabbit cytochrome b_5_ in lipid bilayers. Indicated residues of cytochrome b_5_ interact with DMPC lipid bilayers: {L99}, {W109, W110, W113, L125}, {L121} interact with carbonyls, C_3_ carbons of acyl-chains, and C_4_-C_12_ carbons of acyl-chains, respectively. The average distributions of each domain of the DMPC structures are indicated as PC (the phosphatidylcholine groups), and CG (the carbonyl-glycerol groups). The distance between CG groups is approximately 21.6 Å from the electron density profile for DMPC lipid bilayers in a previous report^93^. The distance between the ^13^C labeled positions of W113 and L125 is 22.4 Å. These two restraints result in the 15° tilt of the transmembrane α-helix relative to the bilayer normal. This orientation of α-helix of cytochrome b_5_ in DMPC lipid bilayers is consistent with our previous studies from oriented ssNMR experiments (~15° ± 3°)^20, 22^.

As mentioned above, the spectra depicted in Figs 1 and 2 display a significant amount of signals from the labeled sites of the protein, in spite of the dominant resonances from natural-abundance ^13^C signals of lipid molecules and glycerol. In order to maintain a physiological membrane environment, the samples in this study contain a large amount of lipids (1:250 protein:lipid molar ratio). However, owing to our strategic labeling scheme, the lipid peaks do not completely overlap with signals from proteins. Thus, well-resolved cross peaks were observed for [3-^13^C] Ala/[2-^13^C] Leu, [2-^13^C] Leu/Trp (indole ring-2-^13^C), [3-^13^C] Ala/[1-^13^C] Val, [2-^13^C] Leu/[1-^13^C] Val, and Trp (indole ring-2-^13^C)/[1-^13^C] Val sites in 2D ^13^C/^13^C PDSD experiments. In addition to these intramolecular ^13^C correlations of cytochrome b_5_ significant lipid-protein intermolecular interactions were observed in the 2D ^13^C/^13^C PDSD chemical shift correlation spectrum, as shown in Fig. 3. It is remarkable that all correlations of natural-abundance ^13^C signals from DMPC lipid in the ^13^C-^13^C PDSD spectra can be readily assigned (see Supplementary Figure S2). Based on these resonance assignments, interatomic interactions between amino acid residues in cytochrome b_5_ (including L99, W109, W110, W113, L121, L125), and DMPC lipid bilayers were unequivocally observed. Specifically, L99 was found to interact with carbonyls of DMPC lipids; W109, W110, W113, and L125 interact with C_3_ carbons of acyl-chains of DMPC lipids; and L121 interacts with C_4_-C_12_ carbons of acyl-chains of DMPC lipids. These experimental results are consistent with previous findings of specific amino acid residues in water-lipid interfaces^85–91^. For example, it is well known that aromatic amino acids, like Trp, can be commonly found at the interfacial domain of the lipid bilayer, where they serve as anchors on the membranes. Particularly, they prefer to locate around the upper chain/glycerol region near C_2_ and C_3_ of acyl chains, due to a multitude of driving forces, including van der Waals interactions, entropic contributions, cation-π interactions, electric dipole interactions, and hydrogen bonding through their imino groups^85, 86^. On the other hand, interaction between L99 and the lipid bilayer also agrees with our previous results obtained by high-resolution MAS NMR and solution NMR on the rabbit cytochrome b_5_ embedded in dodecyl-phosphocholine (DPC) micelles^15, 92^. In the lipid bilayer environment, based on the membrane interaction between the linker region cytochrome b_5_ and the lipid bilayer, it is predicted that the soluble domain of cytochrome b_5_ can adapt certain orientations, which will result in a productive complex formation with cytochrome P450^15, 20, 22^. Particularly, it is known that the soluble domain (particularly, the F-G loop region) of cytochrome P450 is tightly bound to the lipid membrane due to its hydrophobicity; therefore, its redox binding partners, like cytochrome b_5_, have to be suitably oriented to form a productive complex^15, 20, 21^. Thus, restricting the motions of redox partners of cytochrome P450, including cytochrome b_5_ and cytochrome P450-reductase, to produce productive orientations would be one of the biological roles of the relatively flexible linker domain that connects the soluble domain and the transmembrane α-helix domain in the redox partner^15, 92^. In this context, the determination of topologies for each of the structural domains is essential for understanding the physiological functions of bitopic proteins, including membrane-bound cytochromes.

Our experimental results suggest that the transmembrane topology of cytochrome b_5_ can be determined using the aforementioned protein-lipid interactions, as shown in Fig. 3(B). The distance between the ^13^C labeled positions of W113 and L125 is 22.4 Å based on the PDB structure of cytochrome b_5_^15^. In addition, the electron density profile of DMPC lipid bilayers gives a distance of ~21.6 Å between the carbonyl-glycerol groups of lipids in the bilayer^93^. These two restraints result in a 15° tilt of the transmembrane α-helix relative to the lipid bilayer normal. This topological information of the α-helix domain of cytochrome b_5_ in DMPC lipid bilayers is in excellent agreement with our previous ssNMR studies on magnetically-aligned bicelles (15° ± 3°)^20, 22^. This suggests that intermolecular interactions between cytochrome b_5_ and DMPC lipid bilayers, including side chain-lipid interactions, can be retained even under frozen conditions, even though the lipid phases vary from liquid-crystalline phase to gel phase under these two distinct experimental conditions. Furthermore, our previous studies of oriented ssNMR experiments concluded that, upon the full-length cytochrome P450-cytochrome b_5_ complex formation in membrane, the α-helical structure and the topology of the transmembrane domain of cytochrome b_5_ are not altered^20^. In addition, these previous studies indicated a potential direct interaction between the transmembrane domains of cytochromes based on the overall dynamical changes observed; however, specific interaction sites among proteins could not be revealed at atomic-level from these previous studies^20^.

### Revealing intermolecular interactions between cytochromes using DNP sensitivity-enhanced ^13^C-^15^N REDOR-filtered 2D ^13^C/^13^C chemical shift correlation experiments

**Figure 4:**
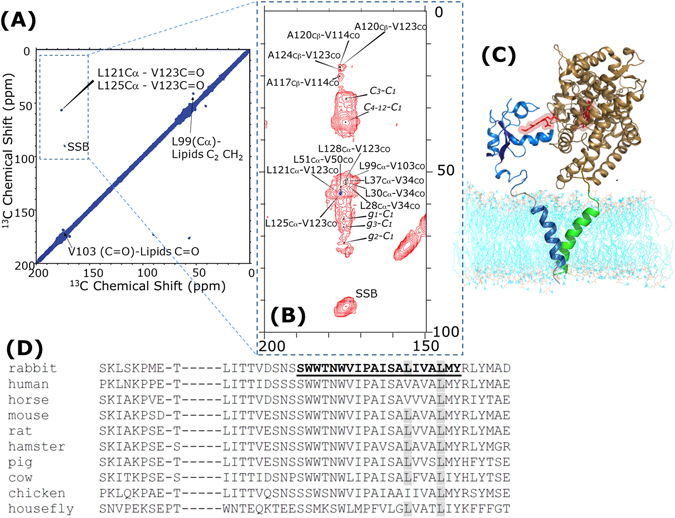
DNP sensitivity-enhanced two-dimensional REDOR-filtered ^13^C-^13^C chemical shift correlation spectrum of selectively ^13^C-labeled cytochrome b_5_-U- ^15^N-labeled cytochrome P450 complex embedded in DMPC MLVs with 10 mM AMUPol in [D_8_]glycerol/D_2_O/H_2_O (60/30/10 volume ratio). **(A)** Natural abundance ^13^C magnetizations of U-^15^N cytochrome P450 selected by ^15^N-^13^C REDOR scheme^104^, which are encoded during the *t*_*1*_ evolution, and subsequently correlated to the neighboring ^13^C nuclei of selectively ^13^C-labeled cytochrome b_5_. The spectrum was obtained with microwave irradiation at 12.5 kHz MAS, 99.5 K sample temperature. A 3 s PDSD mixing time, 256 *t*_*1*_ increments, 128 scans, 32 dummy scans, and 4.68 s recycle delay were used. Total experimental time was 70 hours. The REDOR-filter of 2.56 ms, the CP contact time of 1.5 ms, and 100 kHz SPINAL64 pulse sequence to decouple protons during the signal acquisition of 13 ms. Covariance NMR was used for the two-dimensional spectrum processing. (**B**) DNP sensitivity-enhanced two dimensional ^13^C-^13^C PDSD chemical shift correlation spectra of selectively ^13^C-labeled cytochrome b_5_ embedded in DMPC MLVs from Fig. 2(A) (in red) is overlaid on REDOR-filtered ^13^C-^13^C chemical shift correlation spectrum of selectively ^13^C-labeled cytochrome b_5_-U- ^15^N-labeled cytochrome P450 complex from Fig. 4(A) (in dark blue). **(C**) A structural model of full-length cytochrome P450-cytochrome b_5_ complex is constructed based on transmembrane-transmembrane interactions obtained from DNP-MAS-ssNMR spectroscopy in this study, and our structural model of soluble domain interactions in a previous study^15^. High-resolution NMR structure of full-length rabbit cytochrome b_5_ in lipid bilayers, and the transmembrane α-helix of cytochrome P450 were attained from our previous studies. **(D**) The sequence alignments of cytochrome b_5_ from various eukaryotes suggest the presence of a highly conserved LxxxL motif between L121 and L125. The underlined area is the hydrophobic α-helix transmembrane domain of cytochrome b_5_. Conserved Leucine residues in the position of 121 and 125 are shaded in black. The amino acid sequences and accession numbers obtained from the NCBI nucleotide data bank are rabbit (P00169), human (P00167), horse (P00170), mouse (P56395), rat (P00173), hamster (P70116), pig (P00172), cow (P00171), chicken (P00174), and housefly (P49096).

For decades, it has been widely recognized that the transmembrane domains of cytochrome P450 and cytochrome b_5_ are essential for catalytic activities of cytochrome P450^20–22, 51^. In order to elucidate the physiological roles of these transmembrane α-helices, specifically their roles in molecular interactions, a REDOR-filtered 2D ^13^C/^13^C chemical shift correlation spectrum was obtained, as shown in Fig. 4(A). In this experiment, the natural-abundance ^13^C magnetizations of U-^15^N cytochrome P450 selected by the ^15^N-^13^C REDOR scheme, which are encoded during the *t*_*1*_ evolution, and are subsequently correlated to the neighboring ^13^C nuclei of selectively ^13^C-labeled cytochrome b_5_ during a long PDSD mixing time (~3 s) in order to obtain the ^13^C-^13^C chemical shift correlation shown in Fig. 4 94.

Purified labeled proteins, both rabbit cytochrome b_5_ and cytochrome 2B4, were first assembled into a complex, and then reconstituted into DMPC lipid bilayers hydrated with AMUPol solutions containing [D_8_]glycerol/D_2_O/H_2_O (60:30:10 volume ratio) as described in the materials and methods section. The full-length cytochrome P450 2B4 is known to form the functional complex with the second electron donor, cytochrome b_5_, in lipid bilayer environments as confirmed by the alternation of spin states, termed type I spectral changes under the sample preparation in this study^15, 20, 51^. It is also known that cytochrome P450 is relatively unstable at ambient temperatures; however, the use of DNP spectroscopy under frozen conditions can circumvent this shortcoming^5, 6, 21, 70^. At these cryogenic temperatures, cytochrome b_5_ maintains its topology and α-helical structure in the transmembrane region, hence the functional transmembrane complex of cytochromes can be retained under the experimental conditions employed in this study.

Based on the abovementioned REDOR-filtered ^13^C/^13^C chemical shift correlation experiment, the intermolecular interaction site of the LxxxL motif between L121 and L125 was directly observed by the coherent dipolar-coupling-based ssNMR method, as shown in Fig. 4(A). In addition, protein-lipid interactions, at L99 and V103, were also observed in this spectrum, and they are consistent with observations in our previous reports^15, 92^. These structural constraints from transmembrane-transmembrane interactions obtained from DNP-MAS-ssNMR spectroscopy allow us to construct a structural model for the full-length cytochrome P450-cytochrome b_5_ complex, as shown in Fig. 4(C). In this structural model, high-resolution NMR structure of the full-length rabbit cytochrome b_5_ in lipid bilayers, soluble domain interactions, and the transmembrane α-helix of cytochrome P450 were obtained from our previous studies^15, 21, 92^. According to these studies, the molecular interactions in the soluble domains of cytochromes exhibit dynamic encounter complexes mediated by both hydrophobic and electrostatic interactions with multiple complex formations, which were explained through the mutagenesis studies and line-broadening of unformly-^15^N labeled cytochrome b_5_ using solution NMR spectroscopy^15^. Therefore, the cross peaks from the interactions in soluble domains cannot be observed in our REDOR-filtered 2D spectra. In fact, based on our reported complex structural model in the soluble domain, some labeled positions (*e.g*., Leu41, Ala59, Val66, Ala72, and Leu75) can be close to the cytochrome P450-cytochrome b_5_ complex interface^15^. However, in addition to the dynamic feature of the encounter complexes, these resonances were not observed in the 2D PDSD spectrum (Fig. 2) because (i) the ^13^C labeled sites are too isolated from other labeled sites to correlate, and/or (ii) the cross peaks are in the crowded regions of the two-dimensional spectra. Nevertheless, the labeled sites in this study are useful to effectively assign transmembrane resonances, as already discussed. At the same time, we would like to note that the use of high protein concentration in lipid bilayer samples could complicate the interpretation of observed spectra due to signal from the naturally abundant ^13^C and/or ^15^N nuclei.

Interestingly, the presence of highly conserved LxxxL motif between L121 and L125 was observed in the amino acid sequences of cytochrome b_5_ from various eukaryotes, as shown in Fig. 4(D). The fact that L125 is more preserved in different eukaryotic amino acid sequences might suggest that L125 plays more vital roles in the transmembrane-transmembrane interaction enabled complex formation of cytochromes. Furthermore, the helical wheel diagram of Supplementary Figure S4 suggests that cytochrome P450 2B4 contains many leucine residues in the N-terminal transmembrane α-helix region. Based on these leucine residues present in the transmembrane α-helices of cytochrome b_5_ and cytochrome P450, a *leucine zipper-like* structure can be found in the transmembrane interactions of the cytochrome P450-cytochrome b_5_ complex^33, 40, 82–84^. The experimental results indicate that the topology of cytochrome b_5_ in the DMPC lipid bilayers suggest that amino acid residues, L121 and L125 are located near the upper chain/glycerol region, which has an unique environment for membrane-associated proteins. According to the dielectric structure model of phospholipid bilayers, membrane bilayers possess drastic dielectric gradient distributions. In this near upper chain/glycerol region of DMPC lipid bilayer, the dielectric constant is 20-50. On the other hand, the dielectric constant of water and hydrophobic acyl-chain regions are 78.5 and 2-4, respectively^95, 96^. Thus, hydrophobic interactions between these *leucine zipper-like motifs* near the upper chain/glycerol region, which retains a higher dielectric constant than hydrophobic acyl-chain regions, can be one of the major driving forces that associate the transmembrane α-helices of cytochromes. Herein, possible driving forces for the transmembrane complex formation in cytochrome P450-cytochrome b_5_ would be a combination of: (i) van der Waals-London interactions^89^, (ii) electrostatic attraction, estimated by 1-3 kcal, between oppositely charged electric dipolar moments in the association of C- and N-terminal α-helices^97, 98^, and (iii) hydrophobic interactions of α-helices near the surface of the lipid bilayer based on the *leucine zipper-like motifs* ^33, 40, 82–84^. These types of specific transmembrane dimerization motifs can be found in the amino acid sequences of both cytochrome b_5_ and cytochrome P450; however, the transmembrane region of cytochrome P450 reductase does not possess such type of dimerization motifs^33^. This observation suggests that the roles of transmembrane interaction in the complex formation of cytochrome P450-cytochrome P450 reductase could be different. Such NMR studies of cytochrome P450-cytochrome P450 reductase are currently under investigation in our laboratory.

## Conclusions

This study represents the first experimental report revealing the topology and structure of the transmembrane domains of membrane-bound cytochrome P450-cytochrome b_5_ complex in the functional full-length form. Probing such biologically important protein-protein and lipid-protein interactions of intact bitopic proteins provides an atomic-level understanding of biological functions of hydrophobic transmembrane structures. The robust NMR approach proposed in this study does not, in principle, have limitations on molecular sizes, and can highlight the molecular interactions of membrane proteins between hydrophobic transmembrane domains as well as hydrophilic soluble domains, when isotopically labels strategically placed. We have demonstrated that isotope labeling of proteins at carefully chosen sites can significantly overcome low spectral resolution related difficulties resulting from frozen samples at cryogenic temperatures. Therefore, this methodology of DNP-MAS-ssNMR spectroscopy with a judicious labeling scheme will have a broad impact on atomic-resolution structural studies on a variety of intact bitopic membrane proteins that are unstable, and/or very difficult to produce. Furthermore, numerous membrane proteins such as amyloid peptides, fusion peptides, toxins, natural products and antimicrobial peptides require lower concentrations due to their disruption or permeability of membrane bilayers. For such systems, the biophysical approach proposed in this study would also be greatly advantageous, since only a small amount of sample is required to perform this sensitivity-enhanced DNP-NMR experiment. In particular, the remarkable sensitivity enhancement of NMR signals achieved by DNP at cryogenic temperatures can potentially provide structural information of *in-vivo* membrane protein complexes at-work, as we have recently demonstrated for cytochrome b_5_ in *in-cell* conditions^73, 90^. While tremendous benefits are achieved through the use of sensitivity-enhanced DNP-ssNMR, poor spectral resolution due to frozen molecular motions and/or conformational heterogeneity at cryogenic temperatures is a fundamental bottleneck^70, 72, 99^. Recently demonstrated Overhauser DNP-ssNMR spectroscopy at ambient temperatures could be useful to overcome some of the disadvantages associated with the low spectral resolution of ssNMR spectra^100^. Lastly, we expect the results reported in this study to pave ways for structural and topological investigations of full-length bitopic membrane protein complexes under physiological conditions using sensitivity enhanced DNP-MAS-ssNMR spectroscopy. Additionally, such structural information obtained in cellular settings could potentially provide game-changing insights into their functions, including the design of their novel inhibitors, and drugs^73^.

## Electronic supplementary material

Supplementary Information
