# Supplementary material for: Transmembrane Interactions of Full-length Mammalian Bitopic Cytochrome-P450-Cytochrome-b5 Complex in Lipid Bilayers Revealed by Sensitivity-Enhanced Dynamic Nuclear Polarization Solid-state NMR Spectroscopy
Source: Sci Rep. 2017 Jun 23;7:4116. doi: 10.1038/s41598-017-04219-1 (PMC5482851; doi:10.1038/s41598-017-04219-1)
Supplement: Supplementary file 1 — Supplementary Information [file 41598_2017_4219_MOESM1_ESM.pdf]

SUPPLEMENTARY INFORMATION

Transmembrane Interactions of Full-length Mammalian Bitopic Cytochrome-P450-Cytochrome-b_5 Complex in Lipid Bilayers Revealed by Sensitivity-Enhanced Dynamic Nuclear Polarization Solid-state NMR Spectroscopy

Kazutoshi Yamamoto, Marc A. Caporini, Sang-Choul Im, Lucy Waskell and

*Ayyalusamy Ramamoorthy**

Table of Contents

- **Materials and Methods ... page S2**
- **Optimization of polarizing agents for higher Nuclear Dynamic Polarization (DNP) efficiency of lipid bilayers (Figure S1) ... page S3**
- **Proton T_1 spin-lattice relaxation times obtanined from DMPC lipid bilayers with and without polarizing agents (Table S1) ... page S4**
- **A natural abundance ^{13}C-^{13}C chemical shift correlation spectrum of DMPC lipid bilayers using DNP (Figure S2) ... page S5**
- **Pulse sequences for DNP sensitivity-enhanced MAS ssNMR spectroscopy used to determine intermolecular interactions (Figure S3) ... page S6**
- **Helical wheel projections of transmembrane domains of membrane-bound cytochromes (Figure S4) ... page S7**
- **Assigned chemical shifts of selectively ^{13}C-labeled cytochrome b_5 incorporated into DMPC bilayers (Table S2) ... page S8**
- **Assigned chemical shifts of DMPC bilayers (Table S3) ... page S8**
- **References ... page S9**

Materials and methods

Sample preparation of DMPC multilamellar vesicles with DNP polarizing agents

Stock biradical solutions of [D_8]glycerol/D_2O/H_2O (60:30:10 volume ratio) containing 40 mM DNP polarizing agents (AMUPol,[1] or TOTAPOL[2]) and a DNP solution of [D_8]glycerol/D_2O/H_2O (60:30:10 volume ratio) were prepared, and were kept in a -80°C deep freezer. Twenty five milligrams of DMPC powder was hydrated using stock biradical solutions and/or DNP solution, the resulting samples were homogeneously mixed by vortexing, then freeze-and-thaw cycles were applied five times. Obtained 50%(w/v) DMPC multilamellar vesicles with and without a 10 mM DNP polarizing agent were packed into 3.2 mm sappier MAS rotors. The NMR probe was pre-cooled to 99.5 K before samples were inserted into the probe.

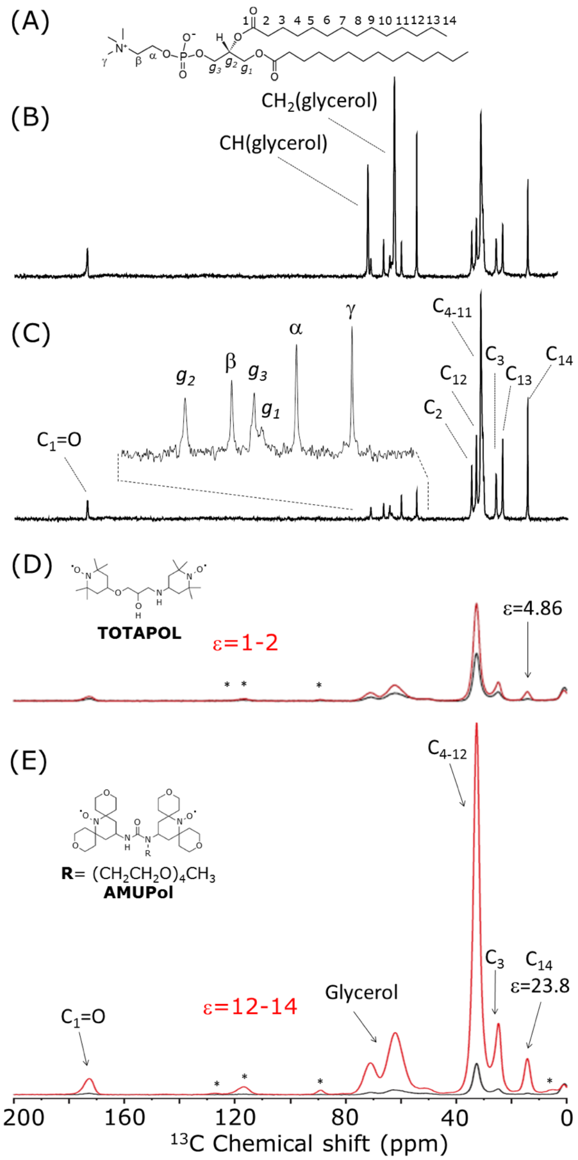

Figure S1. Higher Nuclear Dynamic Polarization (DNP) efficiency of a hydrophilic polarizing agent, AMUPol. (A) The molecular structure of DMPC. One dimensional ^{13}C NMR spectra of 50% (w/v) DMPC mutilamellar vesicles in [D_8]glycerol/D_2O/H_2O (60/30/10 volume ratio) using (B) one pulse excitation, and (C, D, and E) CPMAS[3] with microwave irradiation on (red), and off (black). (D) 10 mM AMUPol, or (E) 10 mM TOTAPOL in [D_8]glycerol/D_2O/H_2O (60/30/10 volume ratio) were used as DNP polarization agents. Magic Angle Spinning speeds were 5.3 kHz (B, and C), and 8.5 kHz (D, and E), and sample temperatures were at 273 K (B, and C) and 99.5 K (D, and E). Recycle delays of 6 s (without DNP polarization agents, B, and C), 1.30 s (for 10 mM TOTAPOL, D), and 2.73 s (for 10 mM AMUPol, E) were used. Total experimental time was 3.33 hours (B, and C), and 3 min for (D, and E). The CP contact times[4] were 5 ms (C), and 0.8 ms (D, and E), and a 50 kHz (B, and C) and 100 kHz (D, and E) SPINAL64 pulse sequence[5] was used for hetero nuclear decoupling during the signal acquisition of 50 ms (B, and C), and 25.9 ms (D, and E). Spinning side bands in the spectra are indicated by asterisks. A background signal arising around 0 ppm is from the silicon rubber seal used in MAS rotors.

	**175.6 ppm (C_1=O)**	**63.8 ppm (Glycerol)**	**27.0 ppm (C_3)**	**16.5 ppm (C_{14})**
**^{1}H T_1 relaxation time of DMPC MLVs with 10 mM AMUPol (s)**	1.98	1.89	2.08	2.05
**ε DMPC MLVs with 10 mM AMUPol**	12.4	13.2	12.5	23.8
**^{1}H T_1 relaxation time of DMPC MLVs with 10 mM TOTAPOL (s)**	0.81	1.93	1.18	0.87
**ε DMPC MLVs with 10 mM TOTAPOL**	1.96	2.06	2.12	4.86
**^{1}H T_1 relaxation time of DMPC MLVs (s)**	30.2	13.7	14.3	N/A

Table S1. Proton T_1 spin-lattice relaxation times obtained from 50%(w/v) DMPC mutilamellar vesicles with and without polarizing agents (10 mM AMUPol, or 10 mM TOTAPOL) in [D_8]glycerol/D_2O/H_2O (60/30/10 volume ratio) using proton T_1 saturation recovery with CP detection, and the signal enhancement (ε) of ^{13}C CPMAS spectra obtained from Figure S1. ^{1}H T_1 spin-lattice relaxation times were measured for selected ^{13}C resonances in DMPC at 8.5 kHz MAS, 99.5 K sample temperature, and 8 scans. NMR spectra was recorded with microwave irradiation for AMUPol/TOTAPOL containing DMPC MLVs. Errors estimated for the reported T_1 relaxation time range from 0.001 to 0.03 s.

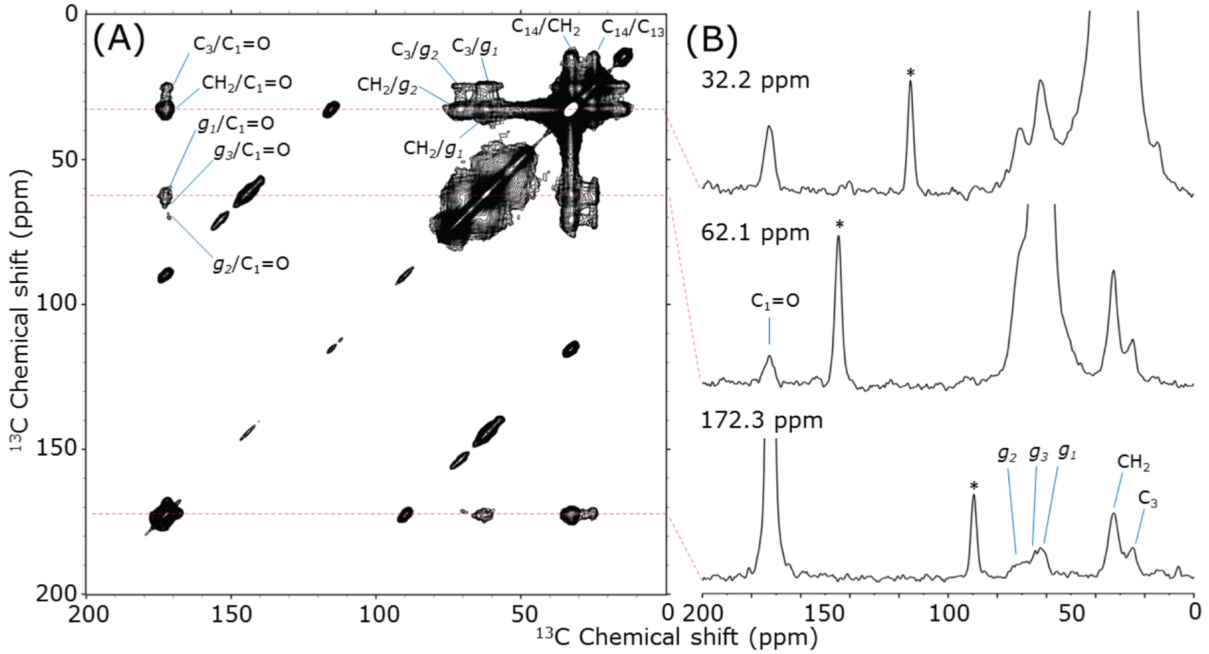

Figure S2. A natural abundance ^{13}C-^{13}C chemical shift correlation spectrum of 50%(w/v) DMPC bilayers via DNP-ssNMR using a high efficient polarizing agent, AMUPol. (A) Two dimensional ^{13}C-^{13}C proton driven spin diffusion (PDSD)[6,7] chemical shift correlation spectrum of DMPC mutilamellar vesicles with 10 mM AMUPol in [D_8]glycerol/D_2O/H_2O (60/30/10 volume ratio) with microwave irradiation at 12.5 kHz MAS, 99.5 K sample temperature. A 3 s PDSD mixing time, 192 t_1 increments, 16 scans, 4 dummy scans, and 2.73 s recycle delay were used. Total experimental time was 4.9 hours. The CP contact time was 1.5 ms and 100 kHz SPINAL64 pulse sequence was used for hetero nuclear decoupling during the signal acquisition of 13 ms. Covariance NMR[8] was used for the two dimensional spectrum processing. (B) One dimensional slices at 32.2 ppm, 62.1 ppm, and 172.3 ppm from two dimensional ^{13}C-^{13}C PDSD chemical shift correlation are shown. Spinning side bands in the spectra are indicated by asterisks.

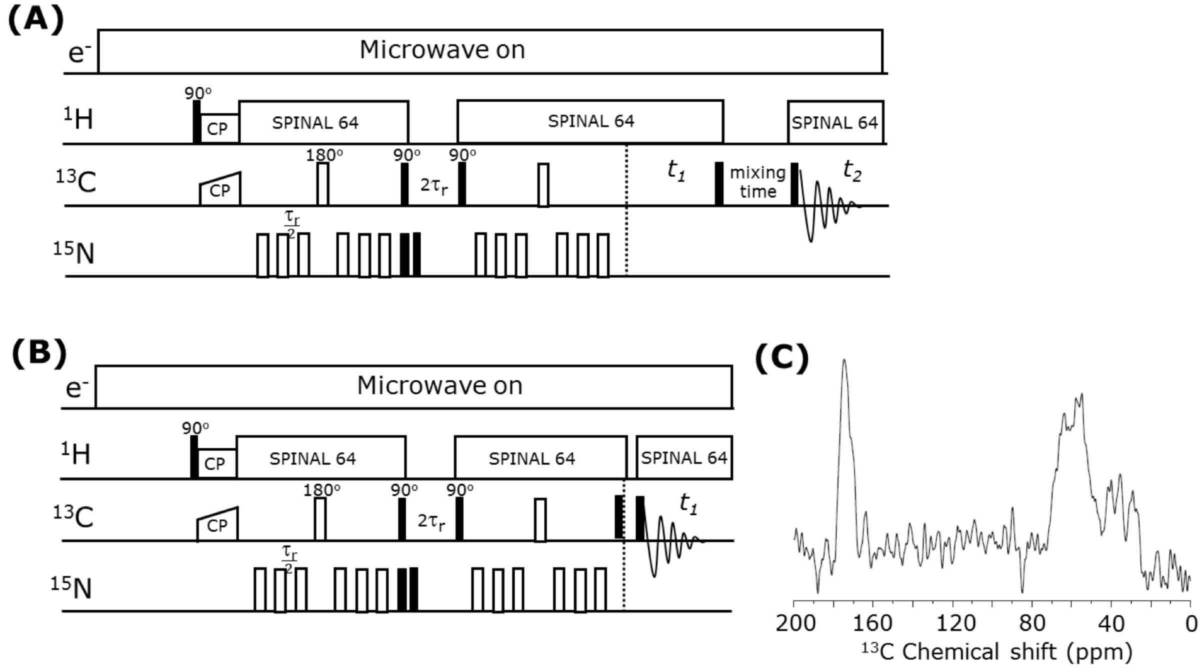

Figure S3. Pulse sequences for DNP sensitivity-enhanced MAS ssNMR spectroscopy used to determine intermolecular interactions between U-^{15}N-labeled cytochrome P450 and selectively ^{13}C-labeled cytochrome b$_5$. (A) Two-dimensional REDOR-filtered ^{13}C-^{13}C chemical shift correlation experiments with microwave irradiation under MAS.[9] The natural abundance ^{13}C magnetizations of U-^{15}N cytochrome P450 selected by ^{15}N-^{13}C REDOR scheme[10], which are encoded during the t_1 evolution, and subsequently correlated to the neighboring ^{13}C nuclei of selectively ^{13}C-labeled cytochrome b$_5$ during a long PDSD mixing time, 3 s, to obtain ^{13}C-^{13}C chemical shift correlation. (B) One-dimensional REDOR-filtered ^{13}C chemical shift experiments. ^{13}C-^{15}N REDOR scheme was used to select the natural abundance ^{13}C resonances located adjacent to ^{15}N spins in U-^{15}N cytochrome P450 using the optimized REDOR-filtering time. (C) One-dimensional REDOR-filtered ^{13}C chemical shift spectrum was obtained using very short 80 μs of PDSD mixing time to prevent ^{1}H spin diffusions, 256 scans, 4 dummy scans, and 4.68 s recycle delay. Total experimental time was 21 minutes. The spectra in both Figure S3(C) and Figure 4(A) were obtained with microwave irradiation at 12.5 kHz MAS, 99.5 K sample temperature, the REDOR-filter of 2.56 ms to select the neighboring natural abundance ^{13}C magnetizations of U-^{15}N cytochrome P450, the CP contact time of 1.5 ms, and 100 kHz SPINAL64 pulse sequence to decouple protons during the signal acquisition of 13 ms.

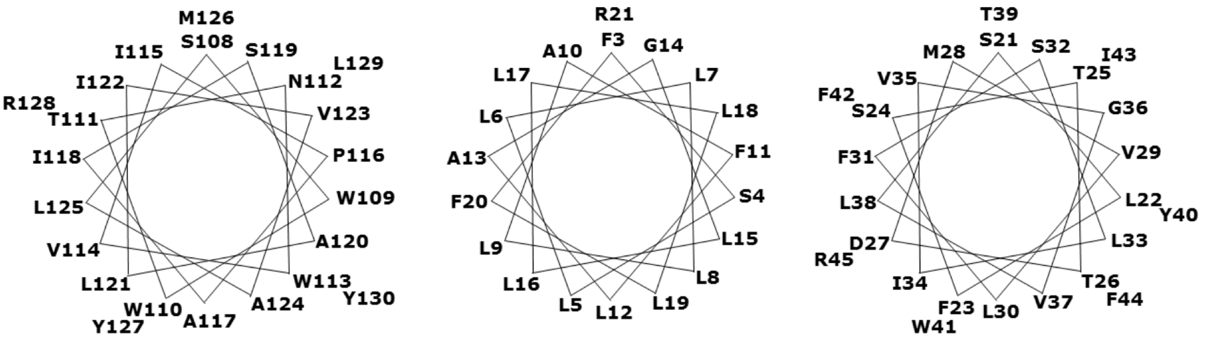

Cytochrome b_5 **Cytochrome P450** **Cytochrome P450 reductase**

Figure S4. Helical wheel projections of transmembrane domains of membrane-bound cytochromes

Amino Acid	Chemical Shift (ppm)
$W27_{C\delta 1}$	126.7
$L28_{C\alpha}$	53.7
$L30_{C\alpha}$	53.2
$V34_{CO}$	174.8
$L37_{C\alpha}$	52.7
$L41_{C\alpha}$	60.0
$V50_{CO}$	176.4
$L51_{C\alpha}$	55.7
$A55_{C\beta}$	20.4
$L99_{C\alpha}$	52.2
$V103_{CO}$	174.3
$W113_{C\delta 1}$	127.5
$V114_{CO}$	176.8
$A117_{C\beta}$	20.3
$A120_{C\beta}$	17.1
$L121_{C\alpha}$	56.7
$V123_{CO}$	176.6
$A124_{C\beta}$	18.5
$L125_{C\alpha}$	57.0
$L128_{C\alpha}$	54.7

Table S2. Assigned chemical shifts of selectively ^{13}C-labeled cytochrome b_5 incorporated into DMPC bilayers.

DMPC	Chemical Shift (ppm)
g_3	67.1
g_2	71.9
g_1	64.6
C_1	175.6
C_3	27.0
$C_{4\text{-}12}$	34.5
C_{14}	16.5

Table S3. Assigned chemical shifts of DMPC bilayers.

References

(1) Sauvée, C. *et al*. Highly efficient, water-soluble polarizing agents for dynamic nuclear polarization at high frequency. *Angew. Chem. Int. Ed. Engl.* **52**, 10858-10861 (2013).

(2) Song, C., Hu, K. N., Joo, C. G., Swager, T. M. & Griffin, R. G. TOTAPOL: a biradical polarizing agent for dynamic nuclear polarization experiments in aqueous media. *J. Am. Chem. Soc.* **128**, 11385-11390 (2006).

(3) Metz, G., Wu, X. & Smith, S. O. Ramped-Amplitude Cross Polarization in Magic-Angle-Spinning NMR. *J. Magn. Reson. A* **110**, 219-227 (1994).

(4) Pines, A., Gibby, M. G. & Waugh, J. S. Proton- Enhanced Nuclear Induction Spectroscopy. A Method for High Resolution NMR of Dilute Spins in Solids. *J. Chem. Phys.* **56**, 1776-1777 (1792).

(5) Fung, B. M., Khitrin, A. K. & Ermolaev, K. An improved broadband decoupling sequence for liquid crystals and solids. *J. Magn. Reson.* **142**, 97-101 (2000).

(6) Bloembergen, N. On the interaction of nuclear spins in a crystalline lattice. *Physica* **15**, 386-426 (1949).

(7) Szeverenyi, N. M., Sullivan, M. J. & Maciel, G. E. Observation of spin exchange by two-dimensional fourier-transform 13C cross polarization-magic-angle spinning. *J. Magn. Reson.* **47**, 462-475 (1982).

(8) Brüschweiler, R. & Zhang, F. Covariance nuclear magnetic resonance spectroscopy. *J. Chem. Phys.* **120**, 5253-5260 (2004).

(9) Wang, T. *et al*. Sensitivity-enhanced solid-state NMR detection of expansin's target in plant cell walls. *Proc. Natl. Acad. Sci.* **110**, 16444-16449 (2013).

(10) Gullion, T. & Schaefer, J. J. Rotational-echo double-resonance NMR. *J. Magn. Reson.* **81**, 196-200 (1989).
